# N-Acetylcysteine Combined With Dexamethasone Treatment Improves Sudden Sensorineural Hearing Loss and Attenuates Hair Cell Death Caused by ROS Stress

**DOI:** 10.3389/fcell.2021.659486

**Published:** 2021-03-18

**Authors:** Xue Bai, Sen Chen, Kai Xu, Yuan Jin, Xun Niu, Le Xie, Yue Qiu, Xiao-Zhou Liu, Yu Sun

**Affiliations:** Department of Otorhinolaryngology, Union Hospital, Tongji Medical College, Huazhong University of Science and Technology, Wuhan, China

**Keywords:** sudden sensorineural hearing loss, hair cell, ROS, N-acetylcysteine, steroid

## Abstract

Sudden sensorineural hearing loss (SSNHL) is a common emergency in the world. Increasing evidence of imbalance of oxidant–antioxidant were found in SSNHL patients. Steroids combined with antioxidants may be a potential strategy for the treatment of SSNHL. In cochlear explant experiment, we found that N-acetylcysteine (NAC) combined with dexamethasone can effectively protect hair cells from oxidative stress when they were both at ineffective concentrations alone. A clinic trial was designed to explore whether oral NAC combined with intratympanic dexamethasone (ITD) as a salvage treatment has a better therapeutic effect. 41 patients with SSNHL were randomized to two groups. 23 patients in control group received ITD therapy alone, while 18 patient s in NAC group were treated with oral NAC and ITD. The patients were followed-up on day 1st (initiation of treatment) and day 14th. Overall, there was no statistical difference in final pure-tone threshold average (PTA) improvement between those two groups. However, a significant hearing gain at 8,000 Hz was observed in NAC group. Moreover, the hearing recovery rates of NAC group is much higher than that in control group. These results demonstrated that oral NAC in combination with ITD therapy is a more effective therapy for SSNHL than ITD alone.

## Introduction

Sudden sensorineural hearing loss (SSNHL) is considered one of the most common emergencies in clinical practice. In the United States, SSNHL is thought to affect between 5–27 in 100,000 individuals, with about 66,000 new cases per year (Alexander and Harris, 2013; Chandrasekhar et al., 2019). In Japan, there are 60.9 cases per 100,000 population diagnosed with SSNHL annually (Nakashima et al., 2014). Further epidemiological investigations have shown that the incidence of SSNHL is increasing globally (Michel, 2011; Kitoh et al., 2020). Currently, high-dose systemic steroid treatment is used as the first-line treatment of SSNHL (Chandrasekhar et al., 2019; Kitoh et al., 2020). However, approximately 50% of patients experience no or limited hearing improvement after systemic steroid treatment (Hunchaisri et al., 2010; Tong et al., 2020). Therefore, those patients with limited hearing improvement (less than 10–20 dB) are considered to have refractory sudden hearing loss (RSHL) (Hunchaisri et al., 2010; Ferri et al., 2012). Although intratympanic dexamethasone (ITD) therapy has been recommended as a salvage treatment for RSHL or after failure of systemic steroid treatment (Moon et al., 2011; Berjis et al., 2016; Sun et al., 2018), its efficacy remains unsatisfactory (Li et al., 2015). It is therefore necessary to devise new strategies for SSNHL.

Steroids combined with another therapy is a common strategy for the treatment of SSNHL. In the United States, hyperbaric oxygen therapy (HBOT) combined with ITD is one option for salvage therapy (Chandrasekhar et al., 2019). Meanwhile, prostaglandin E1 combined with steroids has been recommended by Japanese clinicians for severe to profound SSNHL (Kitoh et al., 2020). Pharmacologically, combination therapy has unique advantages, with potential synergistic effects to achieve better therapeutic outcomes. Recently, antioxidants have been removed from the list of interventions that the American Clinical Practice Guidelines for SSNHL (published in 2019) recommend against using (Chandrasekhar et al., 2019). Although no explanation is given for this change, it indicates that antioxidants may have potential value in the treatment of SSNHL.

To date, a wide variety of antioxidants have been used in the treatment of SSNHL, but their effects remain controversial. Previous studies showed that different combinations or single vitamins (used as antioxidants, vitamin A, C, or E) combined with a steroid were more beneficial for patients with SSNHL (Joachims et al., 2003; Hatano et al., 2008; Kang et al., 2013; Kaya et al., 2015). Similarly, a clinical trial showed that a zinc supplement may enhance the hearing recovery of SSNHL patients by reducing oxidative stress of the cochlea (Yang et al., 2011). However, another study did not find any convincing benefits of a zinc supplement (Niran et al., 2015). Although evidence of an oxidant–antioxidant imbalance was found in SSNHL patients, the therapeutic targets of antioxidants and the mechanism of their interaction with steroids are still difficult to fully elucidate (Jarosław et al., 2019; Ozdamar et al., 2019). Therefore, how to select the effective antioxidant for SSNHL has become a puzzled problem to be solved.

N-acetylcysteine (NAC), as a precursor of glutathione (GSH) and a limiting factor in the process of GSH synthesis, is one of the antioxidants commonly used in the inner ear (Duan et al., 2004; Pathak et al., 2015; Tillinger et al., 2018). It has been clinically proven to be effective as a single therapy in the treatment of SSNHL or cisplatin-induced hearing loss (Riga et al., 2013; Chen and Young, 2016). For initial treatment, combination therapy with corticosteroids plus L-NAC is associated with improved hearing compared to corticosteroids alone (Angeli et al., 2012). Moreover, addition of NAC has been shown to increase glucocorticoid sensitivity in a mouse model of steroid-resistant asthma (Eftekhari et al., 2013). These studies indicate that NAC and steroids may enhance treatment efficacy through synergistic action. Combining a steroid with NAC may be a potential alternative to salvage therapy of SSNHL or RSHL. To prove our hypothesis, *in vitro* experiments were performed to verify whether NAC and steroid have a synergistic effect on oxidative stress injury. In addition, a clinical trial was designed to compare the therapeutic efficacy of ITD with that of ITD combined with NAC in the salvage therapy of SSNHL.

## Materials and Methods

### Culture of Cochlear Explants and Drug Treatments

C57BL/6 mice at P3 were decapitated after anesthesia, then the cochlear basilar membrane was carefully isolated from the cochlea in transparent Hank’s balanced salt solution (PB180321, ProCell, Wuhan, China). The cochlear basilar membrane containing the organ of Corti was transferred onto a collagen gel matrix. A 15 μL droplet of a 9:1:1 rat tail collagen (Type 1-4236, BD Biosciences, Franklin Lakes, NJ, United States), 10 × Basal Medium Eagle (BME; B9638, Sigma-Aldrich, St. Louis, MO, United States), 2% sodium carbonate (P1110, Solarbio, Beijing, China) mixture was placed on the surface of a 35-mm culture dish and allowed to gel for approximately 30 min at 37°C. Afterward, 1.3 mL medium consisting of 1 × BME (41010109, Gibco, Carlsbad, CA, United States) containing 1% bovine serum albumin (A8020, Solarbio), 10% glutamine (G7513, Sigma-Aldrich), 5 mg/mL glucose and 10,000 U/mL penicillin G (P3414, Sigma-Aldrich) were added to the culture dish. The cochlear explants were placed as a flat preparation on the surface of the collagen gel, and the surface of the basilar membrane was exactly even with the culture medium. All explants were incubated overnight at 37°C in an atmosphere of 5% CO_2_. On the following day, the culture medium was removed, the explants of the cochlea for primary culture were treated with fresh medium containing drugs for 24 h *in vitro*, then subjected to immunofluorescent staining. The cochlear explants were divided into four groups and were exposed to 160 U/L glucose oxidase (GO; G3660, Sigma-Aldrich; GO group), 160 U/L GO together with 50 μg/mL dexamethasone (GO + Dex group), 160 U/L GO with 5 mM NAC (A7250, Sigma-Aldrich; GO + NAC group), or 160 U/L GO together with 5 mM NAC and 50 μg/mL dexamethasone (GO + Dex + NAC group). The cochlear explants (*n* = 3–5 in each group) were incubated at 37°C in 5% CO_2_ for 24 h and then harvested for further experiments.

### Cochlear Tissue Preparation and Fluorescent Labeling

The cochlear explants were fixed in 4% paraformaldehyde in 0.01 M PBS for 1 h at room temperature. After washing three times in 0.01 M PBS, explants were stained with DAPI (C1005, Beyotime Institute of Biotechnology, Jiangsu, China) and phalloidin (0.05 mg/mL, P5282, Sigma-Aldrich) for 10 min each. Images were captured with a laser scanning confocal microscope (Nikon, Tokyo, Japan). Three regions from the apical, middle, and basal turns of the stretched cochlear explants were scanned using a ×60 magnification lens.

### Clinical Study Design and Patients

This clinical trial was carried out between March 2017 and March 2019 at the Department of Otorhinolaryngology of Wuhan Union hospital. Eligible subjects were patients with at least 30 dB hearing loss in three contiguous frequencies that had occurred over a course of 3 days, with available previous audiometry data. All patients had a normal otoscopic exam and tympanograms and had not responded to initial treatment. The hearing thresholds of patients were measured at 250–8,000 Hz. Exclusion criteria for the study were: Subjects older than 60 years old (to rule out potential presbycusis); patients with completely hearing loss at 4,000 and 8,000 Hz; patients with Meniere’s disease or other recognized pathologies of SSHL, such as genetic causes, acoustic trauma, previous otologic surgery and so on; any contraindication for the use of NAC and steroids, such as pregnancy or hypertension; MRI scan finding acoustic neuroma or other retrocochlear lesions; disease onset time of more than 14 days; incomplete medical records or inadequate follow-up. All individuals underwent medical history, physical examinations, and laboratory tests, as well as audiologic evaluations that included tympanometry and pure tone audiometry before diagnosis and therapy. All individuals were informed about the procedure and the possible risks. They all agreed to participate in this research and signed an informed consent form. This study was approved by the institutional review board of Wuhan Union hospital.

### Therapy Protocol

After screening for eligibility, all subjects were randomly divided into two groups. Randomization was carried out by generating sequential random numbers using computer-based software. Every recruited individual received sequential random numbers placed in closed envelopes. All doctors and patients were aware of the allocation. The physicians that performed the pure tone audiometry and data analysis were kept blinded to the allocation.

All of the eligible subjects had received ITD injections and basic treatment for SSNHL, which included nourishing nerves and improving vascular microcirculation. All patients underwent hearing tests before treatment and 2 weeks later after treatment. The patients were treated by the senior physicians and received ITD administration alone in the control group. In the experimental groups (NAC groups), all patients routinely received combination therapy with ITD plus oral NAC. NAC (Conbe Biopharmaceutical Company, China) was given orally in the form of effervescent tablets at a dose of 600 mg two times daily for 2 weeks, starting with the with the first IT Dex therapy.

### Measurement of Auditory Function

The audiometric data of all evaluable patients were analyzed. The pure-tone hearing thresholds were measured at 250, 500, 1,000, 2,000, 4,000, and 8,000 Hz. The pure-tone threshold average (PTA) was calculated by measuring the six-frequency average of the threshold value at 500, 1,000, 2,000, and 4,000 Hz. Thresholds that were not measurable because of the limit of the audiometric equipment were coded with the maximum level of the audiometer that was set at 120 dB (HL). Pure tone audiometry was performed before initiation of treatment and 2 weeks after initiation of treatment. The main end-point of this research was the final mean hearing improvement, which was regarded as the difference between initial and final PTA. PTA values were compared to assess the hearing recovery before and after treatment. According to the criteria in the guidelines for the diagnosis and treatment of sudden deafness of the Chinese society of otorhinolaryngology, “hearing improvement” was defined as more than 15 dB hearing gain, and “no improvement” as less than 15 dB hearing gain.

### Cell Culture and Treatment

BxPC3 cells were cultured in high-glucose DMEM (11995500, Gibco) mixed with 5% volume of fetal bovine serum (11054001, Gibco) with antibiotics and incubated in 5% CO_2_ at 37°C. The levels of ROS in cells were detected by staining with dichlorodihydrofluorescein diacetate (DCFH-DA; D6883, Sigma-Aldrich). The cells were exposed to 80 U/L glucose oxidase (GO, GO group) for 4 h. Cells after treatment were washed in pre-warmed PBS and stained with 10 μM DCFH-DA in serum-free DMEM for 30 min. The cell fluorescence intensity was measured by fluorescence microscopy.

### Statistical Analysis

Efficacy was analyzed in all eligible patients. Descriptive statistics were used for the feature description. Paired samples *t* tests were used to compare the means of quantitative variables in the same group at different points in time. Independent samples *t* tests were used to compare the means of metric variables between two groups. Categorial variables were compared using Fisher’s exact test or Chi-square test. A difference was considered to be statistically significant when the *P* value was less than 0.05. All statistical analyses were performed using the SPSS statistical software package (version 22.0; IBM SPSS Statistics for Windows, Armonk, NY, United States). The graphs were created using GraphPad Prism (version 8.2.1).

## Results

### A Cochlear Explant Model of Oxidative Stress Injury Was Established to Verify the Oto-Protective Synergistic Effect of Dexamethasone Combined With NAC

**FIGURE 1:**
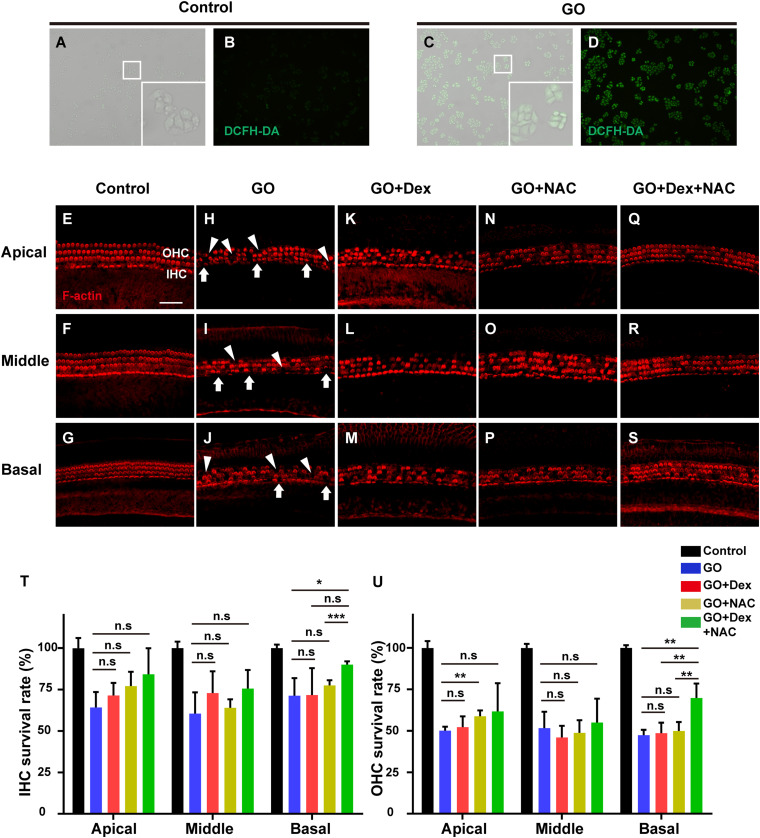
The changes in the number of hair cells after drug treatment for 24 h in ROS models *in vitro* were measured by fluorescence. **(A–D)** The intracellular ROS level in BxPC3 cells was measured in control group and GO group, using a peroxide-sensitive fluorescent probe, DCFH-DA. White boxes in the lower left corner are magnified images. **(E–S)** Representative confocal images showing hair cells from the three turns of the cochlea labeled with F-actin (red) after culturing for 24 h. Images from the control group and the groups treated with GO, GO + Dex, GO + NAC, and GO + NAC + Dex are shown. White arrowheads indicate the missing of hair cells in three turns. **(T)** Comparison of the survival rate of inner hair cells in control, GO, GO + Dex, GO + NAC, and GO + NAC + Dex groups. **(U)** Comparison of the survival rate of outer hair cells in control, GO, GO + Dex, GO + NAC, and GO + NAC + Dex groups. GO, Glucose oxidase; IHCs, inner hair cells, OHCs, outer hair cells; Dex, dexamethasone; NAC, N-acetylcysteine. *P < 0.05, **P < 0.01, ***P < 0.001, and n.s, no significant difference. Scale in panel E represents for 40 μm.

In patients with SSNHL, some indirect evidence of oxidative stress injury has been found successively (Becatti et al., 2017; Jarosław et al., 2019). Therefore, a cochlear explant model of oxidative stress injury was adopted to explore the effects of combined therapy. In the BxPC3 cell line, 4 h of GO treatment significantly increased the intracellular ROS level. The green fluorescent signal in the GO group detected by DCFH-DA, a probe of reactive oxygen species, was much stronger than that of the control group (Figures 1A–D). Therefore, GO was used to increase the ROS level in cochlear explants. Compared to the control group (Figures 1E–G, *n* = 3), a moderate degeneration of hair cells was observed in different turns of the GO group (*n* = 4). After 24 h of GO incubation, half of the outer hair cells (OHCs) had died, while 60.51–71.34% of inner hair cells (IHCs) survived (hair cell loss, white arrows and arrowheads, Figures 1H–J). A low concentration of dexamethasone (50 μg/mL) was added to GO-treated explants (GO + Dex group), and the results revealed that dexamethasone had no protective effect on hair cells at this concentration. The rates of OHC survival were 52.25 ± 6.46, 46.09 ± 6.97, and 48.64 ± 6.32% in the apical, middle and basal turns, respectively (GO + Dex group, *n* = 5). Meanwhile, approximately 70% of IHC survived in the GO + Dex group (Figures 1K–M). Similarly, a non-therapeutic concentration of NAC (5 mM) was also added to the GO-treated explant (GO + NAC group). In this group, the average OHC survival rates were 48.87–58.80% in different turns, while IHC survival rates fluctuated between 64.09 and 77.56% (Figures 1N–P). However, there was a statistically-significant difference in the OHC survival rate of the apical turn between the GO and GO + NAC groups (GO: 50.16 ± 2.36% vs. GO + NAC: 58.50 ± 3.54%, *P* = 0.0042). This improvement of OHC survival was fairly limited (less than 9%). Except for the above improvement, there was no statistically-significant difference in hair cell survival rates between the GO + NAC group and the GO group (Figures 1T,U). When Dex and NAC were both added to GO-treated cochlear explants (*n* = 5), the number of surviving IHCs or OHCs in the basal turn was significantly increased (Figures 1Q–S). Compared with the GO group, the survival rates of OHC and IHC in the basal turn of the GO + Dex + NAC group were significantly increased (OHC, 47.41 ± 3.20 vs. 69.85 ± 8.65%, *P* = 0.0018; IHC, 71.34 ± 10.56 vs. 90.00 ± 2.00%, *P* = 0.0057, Figures 1T,U).

### Clinical Trial to Observe the Effectiveness of NAC Combined With Dexamethasone Therapy

**FIGURE 2:**
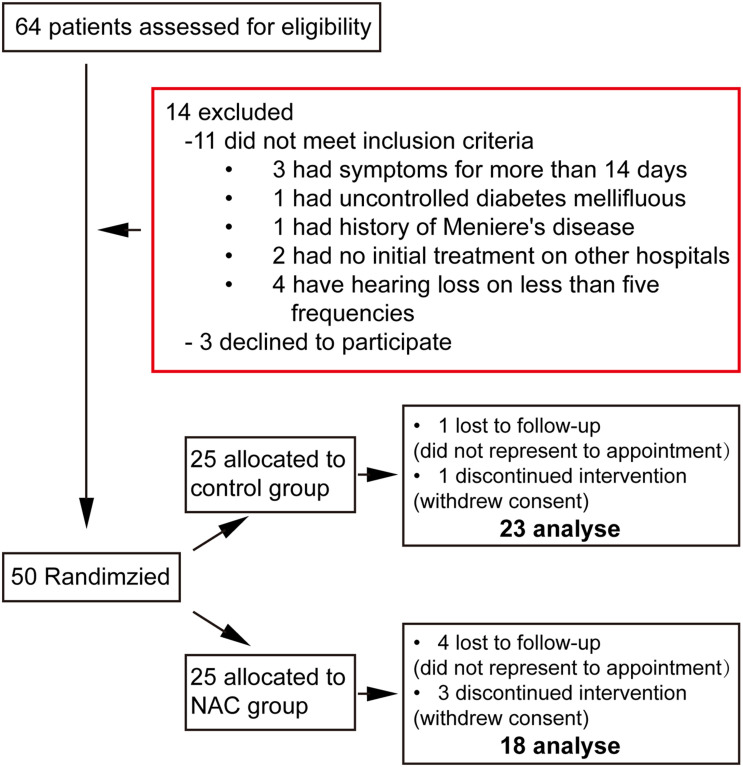
Study flowchart.

There was a total of 64 patients who agreed to take part in this study. Of those, 14 were excluded for not conforming to eligibility criteria, consisting of three patients who had experienced symptoms for more than 14 days, one who had uncontrolled diabetes mellitus, one patient with a history of Meniere’s disease, two patients who had not undergone any initial treatment at other hospitals, four patients who did not have hearing loss in five frequencies, and three participants who declined to take part. The remaining 50 patients who agreed to participate were randomized into two groups for further treatment and analysis. Of the 50 participants included, five were later excluded because of loss of contact from this clinical trial, and four patients were excluded owing to withdrawal of consent. Finally, overall 41 patients were included in our analysis. There were 23 patients in the control group, while 18 patients were analyzed in the NAC group (Figure 2).

All 41 patients were recruited at the Department of Otorhinolaryngology of Wuhan Union hospital. Sixteen of the patients were male (39.0%) and 25 were female (61.0%) with an average age of 38.5 ± 14.4 years (range: 14–60). Twenty-three (56.1%) participants were randomized to the control group, and 18 (43.9%) to the NAC group. The initial PTA of all patients was 64.1 ± 21.5 dB HL (66.25 ± 20.17 dB in the control group, 61.3 ± 22.8 dB in the NAC group). The post-treatment PTA of the control group was 54.7 ± 25.4 dB HL, while it was 42.2 ± 26.3 dB HL in the NAC group. For all patients, the mean duration of hospital treatment was 11.8 ± 4.7 days (11.8 ± 4.9 days in the control group; 12 ± 4.5 days in the NAC group). The mean PTA gain of all patients was 14.9 ± 15.5 dB (11.6 ± 17.8 dB in the control group; 19.0 ± 11.1 dB in the NAC group). No statistically-significant differences were found between the control and NAC groups concerning age, sex, days in hospital, initial PTA, final PTA, or mean PTA gain (*P* > 0.05, Table 1).

**TABLE 1:** Demographic and audiological features of patients in control group and NAC group.

	All (*n* = 41)	Control(*n* = 23)	NAC(*n* = 18)	*P* value
Age (years) (mean ± SD)	38.54 ± 14.35	41.96 ± 12.50	34.17 ± 15.69	0.08
Gender (male/female)	16: 25	8: 15	8: 10	0.54
Time in hospital (days) (mean ± SD)	11.88 ± 4.66	11.78 ± 4.88	12.00 ± 4.50	0.88
Initial PTA (dB) (mean ± SD)	64.05 ± 21.52	66.25 ± 20.72	61.25 ± 22.78	0.46
Final PTA (dB) (mean ± SD)	49.21 ± 26.20	54.67 ± 25.37	42.22 ± 26.26	0.24
Mean PTA gain (Db) (mean ± SD)	14.85 ± 15.54	11.57 ± 17.84	19.03 ± 11.09	0.17
ALT (U/T) (mean ± SD)	23.63 ± 16.08	23.52 ± 14.41	23.78 ± 18.43	0.96
Blood fat (Dyslipidemia/Ortholiposis)	19: 22	10: 13	9: 9	0.65
NLR (mean ± SD)	2.22 ± 1.16	2.12 ± 0.74	2.34 ± 1.57	0.57

*PTA, pure tone audiometry; ALT, alanine transaminase; NLR, neutrophil-to-lymphocyte ratio; SD, standard deviation; NAC, N-acetylcysteine. PTA pure tone average at 500, 1,000, 2,000, and 4,000 Hz hearing thresholds.*

**FIGURE 3:**
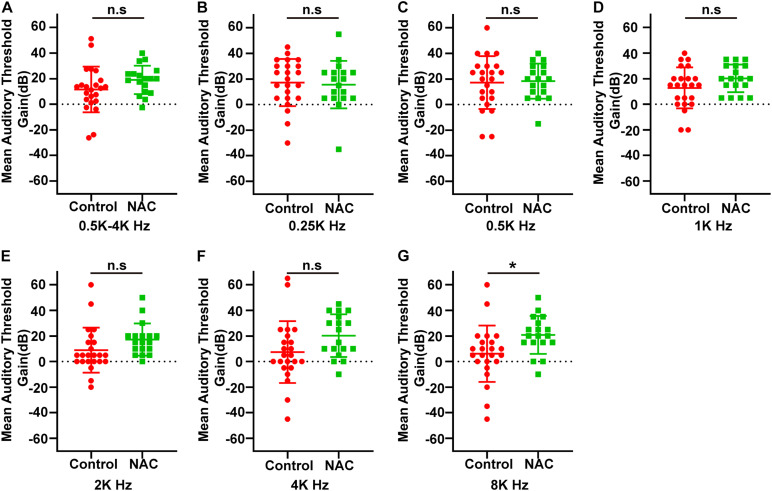
**(A)** The mean hearing gain at 0.5–4k Hz in control and NAC groups. **(B–G)** The hearing gain at 0.25k **(B)**, 0.5k **(C)**, 1k **(D)**, 2k **(E)**, 4k **(F)**, and 8k **(G)** Hz in two groups. *P < 0.05 and n.s, no significant difference.

Figure 3 indicates the audiologic outcomes at different frequencies for all patients in the control and NAC groups. The hearing gains of the control group were 17.2 ± 18.5, 17.2 ± 20.7, 12.8 ± 16.1, 8.9 ± 17.6, 7.4 ± 24.1, and 6.1 ± 22.1 dB at 250, 500, 1,000, 2,000 4,000, and 8,000 Hz, respectively. These gains in the NAC group were 15.6 ± 18.5, 18.3 ± 13.7, 20.3 ± 10.8, 17.2 ± 12.6, 20.3 ± 16.6, and 20.8 ± 14.8 dB, respectively, at the corresponding frequencies. Compared with the control group, the mean gain of the NAC group was significantly different at 8,000 Hz (*P* = 0.019, Power = 0.854, Figure 3G). No statistically-significant differences were detected at any of the other frequencies (*P* > 0.05, Figures 3A–F).

In terms of hearing recovery (Table 2), any PTA gain (500–4,000 Hz) greater than 15 dB is considered effective. The percentage of patients who experienced effective recovery in the control group vs. the NAC group was 39.1% (9 of 23) vs. 72.2% (13 of 18). Thus there was a significant difference in the effective rate between control and NAC groups (*P* = 0.035, Pearson’s chi-squared test).

**TABLE 2:** Hearing improvements, respectively, in control and NAC group.

	Control group (*n* = 23)	NAC group (*n* = 18)
Hearing improvement (%)	9 (39%)	13 (72%)
No improvement (%)	14 (61%)	5 (28%)

**P* = 0.035.*

## Discussion

Hair cells mainly function in transducing sound waves into the electric signals (Wang Y. et al., 2017; Liu Y. et al., 2019; Qi et al., 2019). Hearing loss could be caused by genetic factors, aging, chronic cochlear infections, ototoxic drugs, and noise exposure (Zhu et al., 2018; Zhang Y. et al., 2019; Zhou et al., 2020). The reported mechanisms of hair cell damage mainly include mechanical shearing forces and oxidative damage to HCs (Liu et al., 2016; He et al., 2017; Li et al., 2018; Zhong et al., 2020), eventually induce apoptotic cell death in HCs, especially the outer HCs of the basal turn. The loss of sensory hair cells is irreversible in adult mammals. Although the neonatal cochlea has very limited hair cell regeneration ability, this regeneration ability is rapidly reduced with age (Wang et al., 2015; Zhang S. et al., 2019, 2020; Zhang Y. et al., 2020). It is still a controversy that the mechanism of SSNHL in the cochlea, Capaccio et al. (2012) had done a research indicating that the patients in SSNHL group had higher serum levels of ROS than in control group, therefore, researcher speculated that hearing loss in SSNHL may be due to antioxidant system failing to handle a sudden rise in ROS. There were studies indicating that excessive ROS, which was produced by noxious stimulation (such as noise, drug) in the cochlea, can destroy hair cell components by oxidizing molecules, such as DNA, proteins (Fechter, 2005; Li and Steyger, 2009). And the unbalance of antioxidant system can activate the programmed cell death pathway in cochlea, causing sensorineural hearing loss (Liu et al., 1998). Therefore, the GO model, a classical oxidative stress model, was used to set up a cochlear explant model of oxidative stress injury for studying SSNHL *in vitro* in our study. Our data show that NAC and dexamethasone have an obvious synergistic effect in the treatment of hair cell damage induced by oxidative stress *in vitro*. There is plenty of evidence to suggest that NAC used as an antioxidant can attenuate hair cell degeneration or deafness *in vitro* or in different animal models of deafness (Kopke et al., 2000; Ohinata et al., 2003; Duan et al., 2004; Wang W. et al., 2017; Liu W. et al., 2019). Moreover, glucocorticoids have strong anti-inflammatory, anti-toxic immunoregulatory effects. Some studies have reported that glucocorticoids can protect hair cells from a variety of adverse factors, such as noise and inflammation (Hirose et al., 2007; Haake et al., 2015; Müller et al., 2017). Evidence from animal and *in vitro* experiments suggests that hair cell protection may be one of the common therapeutic effects of NAC and glucocorticoid. In our *in vitro* experiments, the combination of NAC and glucocorticoids was effective in protecting hair cells, although they were administered at concentrations which were ineffective when used alone. Previous research suggests that NAC may be a steroid sensitizer which can help to treat steroid-resistant asthma in mice (Eftekhari et al., 2013). At present, we do not know whether the protection of hair cells is caused by NAC increasing the therapeutic effect of dexamethasone. However, this interesting finding has potential value for clinical application. It may help us overcome steroid-resistant SSNHL or achieve better results with smaller doses of drugs.

N-acetylcysteine combined with ITD therapy can significantly improve hearing loss at high frequency in SSHNL patients. A study by Machado et al. showed that ITD combined with oral prednisone and NAC can improve hearing loss at 4,000 Hz as initial therapy. However, it was not until 6 months later that there was a statistically-significant difference between the steroid alone group and the steroids + NAC group (Angeli et al., 2012). In our study, the onset of hearing loss of all patients was at more than 2 weeks. Therefore, a salvage therapy of ITD was adopted without oral steroids. Considering the safety of the drug, the oral dose of NAC was 600 mg two times daily in our design, which was half of the dose used in the study by Machado et al. Since we observed the protection of hair cells in the high frequency region *in vitro*, all the patients chosen in this study suffered hearing loss in the high frequency region (4,000 or 8,000 Hz). Our data showed that ITD combined with NAC as a salvage approach was found to significantly improve high-frequency hearing loss in patients with SSHNL.

The protective effect at high frequency may be caused by increasing the sensitivity of the inner ear to dexamethasone through oral NAC. When ITD is used in clinical practice, both injection dose and interval may affect the efficacy (Liebau et al., 2016). Recent studies found that the therapeutic effect of steroids on SSNHL can be significantly improved through use of a microcatheter with an electronic pump, near-continual transtympanic steroid perfusion or ITD administration using saturated Gelfoam (Chou et al., 2013; Li et al., 2013; Lundy et al., 2018). The above evidence suggested that increasing the amount and the effective time of steroids in the inner ear can significantly improve the therapeutic effect on SSNHL. However, the above method requires a complicated operation and corresponding equipment, making it difficult to popularize at present. Our finding, from another perspective, can achieve similar goals. We speculate that oral NAC may increase the effectiveness of steroids by reducing the minimum effective concentration or extending the effective duration. However, NAC may also help the hearing recovery of SSNHL patients through direct antioxidant effects.

Although there were some interesting findings, our study does have some limitations. Firstly, significant hearing improvement occurred only at high frequency. We and Machado et al. failed to observe any effect of this combination therapy on hearing improvement at low frequencies. Different doses of NAC and different methods of administration may need to be tried in the future. Secondly, the specific therapeutic mechanism of this combined therapy remains unknown. Although one study suggested that NAC alone can improve SSNHL, our *in vitro* experiment indicated that the synergistic effect of NAC and steroids may play a key role in protecting hair cells from oxidative stress. More evidence is needed to determine whether antioxidant therapy alone is effective against SSHNL. Finally, in our clinical trial, it was difficult to perform subgroup analysis of the different deafness types due to the limited numbers of patients. More extensive studies related to the associations between NAC and subtypes of SSNHL will be conducted with larger numbers of patients in future.

## Conclusion

In this study, we found that NAC combined with dexamethasone may protect against hearing damage by protecting hair cells. The results of our clinical study suggest that the use of NAC in combination with ITD is beneficial in the 8,000 Hz frequency. Combined therapy of NAC and ITD can improve the hearing recovery rate of patients with SSNHL.

## Data Availability Statement

The raw data supporting the conclusions of this article will be made available by the authors, without undue reservation.

## Ethics Statement

The studies involving human participants were reviewed and approved by the institutional review board of Wuhan Union hospital. Written informed consent to participate in this study was provided by the participants’ legal guardian/next of kin. The animal study was reviewed and approved by the institutional review board of Wuhan Union hospital. Written informed consent was obtained from the owners for the participation of their animals in this study.

## Author Contributions

YS and SC conceived and designed the experiments. XB and KX performed the experiments *in vitro*. XB and XN collect the clinic data. YJ, LX, and YQ analyzed the data. XB, SC, and YS wrote the manuscript. All authors contributed to the article and approved the submitted version.

## Conflict of Interest

The authors declare that the research was conducted in the absence of any commercial or financial relationships that could be construed as a potential conflict of interest.
